# Supplementary material for: Cortical microstructural associations with CSF amyloid and pTau
Source: Mol Psychiatry. 2023 Dec 13;29(2):257–68. doi: 10.1038/s41380-023-02321-7 (PMC11116103; doi:10.1038/s41380-023-02321-7)
Supplement: Supplementary file 1 — Supplementary Appendix [file 41380_2023_2321_MOESM1_ESM.pdf]

SUPPLEMENTARY APPENDIX

Cortical microstructural associations with CSF amyloid and pTau

Table of Contents

1. Supplementary Methods 3
1.1 Description and interpretation of MAP-MRI measures 3
Supplementary Table 1
1.2 Cortical dMRI measure extraction flow chart 4
Supplementary Figure 1
1.3 MT-NODDI parallel diffusivity MSE comparisons 5
Supplementary Figure 2
1.4. Proposed mediation analysis 5
Supplementary Figure 3
2. Supplementary Results 6
2.1 Characteristics of study participants by diagnostic group 6
Supplementary Table 2
2.2 Correlation between cortical AD-metaROI measures 7
Supplementary Figure 4
2.3 CSF biomarker associations with cortical MRI measures 8
Supplementary Table 3
2.4 Log10-transformed $pTau_{181}$ associations 8
Supplementary Table 4
Supplementary Figure 5
2.5 Sensitivity analysis: CSF biomarker associations in non-demented participants 10
Supplementary Table 5
Supplementary Figure 6
Supplementary Figure 7
Supplementary Figure 8
2.6 Clinical diagnosis differences in cortical MRI measures 14
Supplementary Table 6
Supplementary Figure 9
2.7 AD-metaROI MRI differences between CSF and clinical diagnosis group 16
Supplementary Figure 10
Supplementary Figure 11
2.8 Interactive effects of CSF biomarker on AD-metaROI MRI measures 18
Supplementary Figure 12
Supplementary Figure 13
2.9 Exploratory CSF biomarker group classification by cortical AD-metaROI measures 19
Supplementary Table 7
Supplementary Figure 14
Supplementary Table 8
Supplementary Figure 15
Supplementary Table 9
Supplementary Figure 16
Supplementary Table 10
2.10 Sensitivity analysis: Mediation analyses in non-demented participants 24
Supplementary Table 11

2.11 ADSP-PHC cognitive domain mediation analyses **25**
Supplementary Table 12
Supplementary Table 13
2.12 Spatially distinct patterns of ICVF and ISOVF associations with amyloid **26**
Supplementary Figure 17
2.13 CSF pTau and delayed working memory **29**
Supplementary Figure 18
3. Supplementary Reference **30**

1. Supplementary Methods

1.1 Description and interpretation of MAP-MRI measures

Supplementary Table 1. Description and interpretation of MAP-MRI measures (1). We note that MAP-MRI is a signal-based model and measures therefore lack specificity; interpretations identified below are biological correlates of each measure identified through simulation or histological validation studies.

**MAP-MRI Measure**		**Description**	**Interpretations**
**MSD**	Mean Square Displacement	Measure of the distance water protons are able to diffuse on average during the diffusion time (more sensitive to larger displacements/more Gaussian/fast moving water molecules) (2)	● Similar to DTI-MD, reflects surface-to-volume ratio of cellular membranes<br>● Sensitive to average cellular density and cytoarchitecture heterogeneity in the cortical GM (2)
**QIV**	Q-Space Inverse Variance	Pseudo-diffusivity measure that is sensitive to the heterogeneity of the speed of diffusion (i.e., the diffusivity). It directly estimates the inverse of the "variance" of the diffusion signal. If the diffusion is Gaussian, it will be identical to MSD. In non-Gaussian diffusion it will be sensitive to the slow diffusion component present within restricted spaces	● Similar to MSD, it is sensitive to the average cellular density, but especially to the heterogeneity of cellular components in the tissue (3). This is shown by the larger intensity contrast between WM/GM/CSF present in QIV and not in MSD. High grade gliomas with increased cellularity and heterogeneity in cellular morphology have been strongly correlated with QIV (4)<br>● Particularly sensitive to variance in diffusion in regions with low restriction volume fractions like the GM (5)
**RTOP**	Return-to-origin Probability	Probability that water molecules undergo zero net displacement (i.e., diffusion is restricted by barriers)	● Reflects presence of restrictive barriers<br>● Sensitive to pore volume (e.g., axonal density in WM((6)<br>● Correlated with number of axons, myelin volume fraction, and fraction of restricted water in the WM (5-7)
**RTAP**	Return-to-axis Probability	Probability that water molecules undergo zero net displacement along the direction of primary eigenvector (i.e., variant of RTOP decomposed along the direction of primary eigenvector)	● Reflects presence of restrictive barriers in the axial orientation<br>● Sensitive to pore cross sectional area (e.g., axonal diameter) (5, 6)<br>● Decreases with dispersion and crossing fibers; particularly sensitive in anisotropic WM (5, 6)<br>● Presents higher contrast than RTPP at the WM-GM boundary (6)<br>● Sensitive to restricted volume fraction in GM (5)
**RTPP**	Return-to-plane Probability	Probability that water molecules undergo zero net displacement perpendicular to direction of primary eigenvector (i.e., RTOP variant decomposed perpendicular to primary eigenvector direction)	● Reflects presence of restrictive barriers in the radial orientation<br>● Increases with dispersion (6)<br>● Shows low contrast between GM and superficial WM since the diffusion restricted along the main diffusion direction is not modeled (only perpendicular); however, shows higher values in fiber crossing regions (6)

1.2 Cortical dMRI measure extraction flow chart

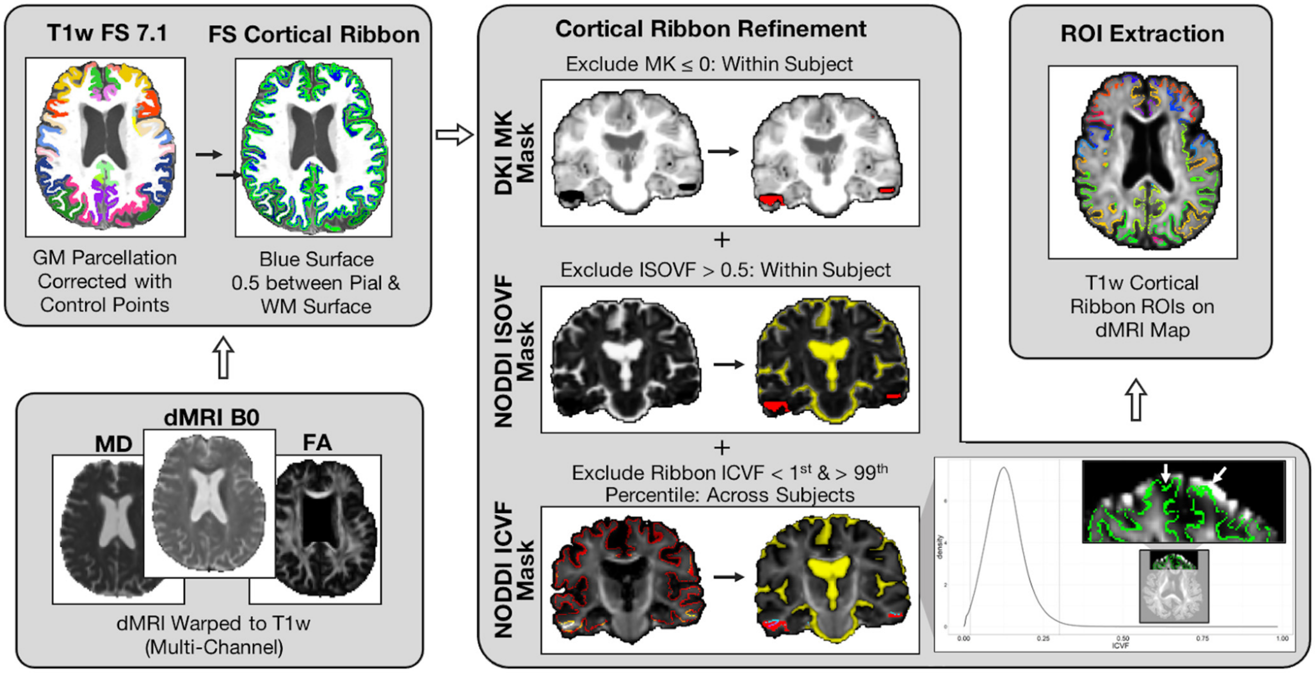

Supplementary Figure 1. After spatially normalizing dMRI maps to respective parcellated T1w images, mean dMRI measures were extracted from a cortical ribbon halfway between the pial and white matter surfaces. To further mitigate any remaining artifacts, partial volume effects, and misregistration the following were excluded from the cortical ribbon: (1) voxels with implausible negative kurtosis values, i.e., DKI MK value ≤ 0; (2) voxels with CSF partial voluming, i.e., ISOVF > 0.5; and (3) as illustrated, voxels in the ribbon that had extreme ICVF values for the sample, i.e., voxels with ICVF values in the 1st and 99th percentiles across all participants. These tended to occur in regions with uncorrected EPI induced distortions such as the frontal and temporal lobes.

1.3 MT-NODDI parallel diffusivity MSE comparisons

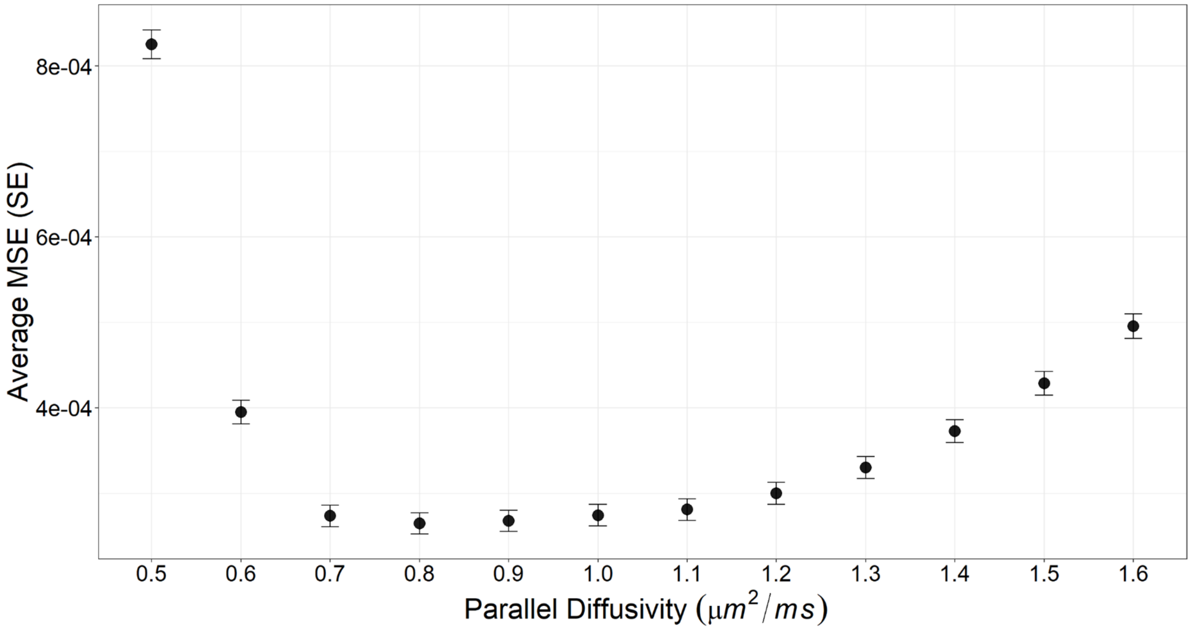

Supplementary Figure 2. The average mean square error (MSE) for each $d_{\parallel}$ between MT-NODDI measured and predicted signals averaged within the medial cortical ribbon across CU participants. The lowest error was found with $d_{\parallel} = 0.8$ µm^2/ms. Paired two-sided t-tests revealed cortical MSE for $d_{\parallel} = 0.8$ was significantly lower than all other $d_{\parallel}$ values.

1.4 Proposed mediation analysis

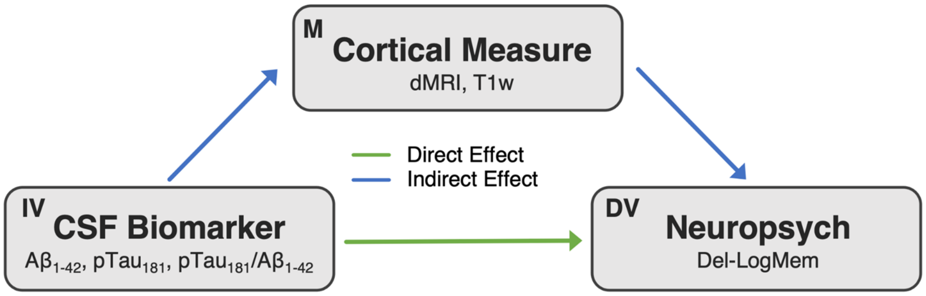

Supplementary Figure 3. Proposed mediation analysis. *Key: IV: Independent Variable; M: Mediator; DV: Dependent Variable.*

2. Supplementary Results

2.1 Characteristics of study participants by diagnostic group

Supplementary Table 2. Basic demographic and clinical characteristics of study participants by diagnostic group, i.e., cognitively unimpaired (N=46) or impaired (N=18 MCI and N=2 dementia). ***Key:*** *MMSE: Mini-Mental State Examination (8); CDR-SoB: Clinical Dementia Rating Scale sum-of-boxes (9).*

		CSF Aβ or pTau Group				CSF pTau/Aβ Ratio Group		CSF Aβ and pTau Group			
	**Total**	**Aβ+**	**Aβ-**	**pTau+**	**pTau-**	**pTau/Aβ +**	**pTau/Aβ -**	**Aβ-/pTau-**	**Aβ-/pTau+**	**Aβ+/pTau-**	**Aβ+/pTau+**
**Cognitively Unimpaired Participants**											
**N**	46	17	29	13	33	11	35	22	7	11	6
**Age, yrs (SD)**	71.6 (5.9)	72.9 (4.8)	70.8 (6.5)	72.8 (7.3)	71.1 (5.4)	76.0 (5.9)	70.2 (5.3)	70.8 (5.6)	71.7 (9.1)	71.7 (5.0)	75.1 (3.8)
**Sex, N Male**	12	5	7	3	9	2	10	6	1	3	2
**APOE4[1], N carriers**	16<br>N=45	7<br>N=16	9	5<br>N=12	11	5<br>N=10	11	7	2	4	3<br>N=5
**Education, yrs (SD)**	16.5 (2.4)	16.6 (4.8)	16.4 (6.5)	15.5 (2.3)	16.9 (2.3)	16.4 (2.3)	16.5 (2.3)	16.7 (2.5)	15.7 (2.3)	17.4 (1.9)	15.2 (2.6)
**MMSE (SD)**	29.0 (1.1)	29.0 (1.2)	28.9 (1.1)	29.2 (1.2)	28.9 (1.1)	29.0 (1.3)	28.9 (1.1)	28.8 (1.1)	29.3 (1.0)	29.0 (1.2)	29.0 (1.3)
**CDR-SoB (SD)**	0 (0)	0 (0)	0 (0)	0 (0)	0 (0)	0 (0)	0 (0)	0 (0)	0 (0)	0 (0)	0 (0)
**Delayed Logical Memory (SD)**	14.1 (4.0)	14.5 (3.8)	13.8 (4.2)	13.6 (4.2)	14.2 (4.2)	14.1 (3.6)	14.1 (4.2)	13.8 (3.9)	14.0 (5.3)	15.2 (4.2)	13.2 (2.8)
**CSF, pg/mL (SD)**											
**$A\beta_{1-42}$**	1204.1<br>(432.6)	719.0 (176.7)	1488.2<br>(237.7)	1139.9 (487.8)	1229.2 (414.3)	695.0 (241.1)	1363.9 (347.1)	1474.9<br>(246.4)	1530.1<br>(220.0)	737.8 (152.6)	684.6<br>(226.1)
**$pTau_{181}$**	20.8 (9.4)	21.4 (12.2)	20.5 (7.5)	32.2 (10.0)	16.3 (3.7)	30.0 (13.4)	18.1 (5.6)	17.3 (4.0)	30.6 (8.2)	14.4 (4.0)	24.1 (12.4)
**$pTau_{181}/A\beta_{1-42}$**	0.021 (0.016)	0.033 (0.021)	0.014 (0.006)	0.036 (0.023)	0.015 (0.007)	0.044 (0.019)	0.014 (0.003)	0.012<br>(0.002)	0.021<br>(0.009)	0.021 (0.009)	0.054<br>(0.021)
**Cognitively Impaired Participants**											
**N**	20	12	8	9	11	12	8	5	3	6	6
**Age, yrs (SD)**	72.4 (7.0)	73.2 (6.4)	71.1 (8.1)	71..9 (7.6)	72.8 (6.9)	73.2 (6.4)	71.1 (8.1)	72.4 (9.9)	68.9 (4.8)	73.1 (4.0)	73.4 (8.7)
**Sex, N Male**	13	9	4	7	6	9	4	2	2	4	5
**APOE4[1], N carriers**	11<br>N=18	10<br>N=11	1<br>N=7	6<br>N=8	5<br>N=10	10<br>N=11	1<br>N=7	0<br>N=4	1	5	5<br>N=5
**Education, yrs (SD)**	15.6 (3.0)	15.3 (3.3)	16.0 (2.6)	14.9 (3.2)	16.1 (2.9)	15.3 (3.3)	16.0 (2.6)	16.8 (2.3)	14.7 (3.1)	15.5 (3.4)	15.0 (3.5)
**MMSE (SD)**	26.3 (3.4)	25.2 (3.4)	28.0 (2.9)	25.4 (4.0)	27.0 (2.8)	25.2 (3.4)	28.0 (2.9)	27.2 (3.5)	29.3 (1.2)	26.8 (2.5)	23.5 (3.4)
**CDR-SoB (SD)**	2.2 (1.5)	2.5 (1.1)	1.9 (2.0)	3.2 (1.6)	1.4 (0.9)	2.5 (1.1)	1.9 (2.0)	1.0 (0.9)	3.3 (2.8)	1.8 (0.8)	3.2 (1.0)
**Delayed Logical Memory (SD)**	5.9 (4.6)	3.8 (4.7)	8.9 (2.5)	4.3 (4.6)	7.1 (4.5)	3.8 (4.7)	8.9 (2.5)	8.8 (2.2)	9 (3.6)	5.7 (5.6)	2 (3.0)
**CSF, pg/mL (SD)**											
**$A\beta_{1-42}$**	876.7 (524.5)	502.4 (196.4)	1438.1<br>(292.5)	830.8 (463.3)	914.2 (589.5)	502.4 (196.4)	1438.1 (292.5)	1483.6<br>(315.2)	1362.3<br>(294.8)	439.98<br>(145.3)	565.0<br>(233.2)
**$pTau_{181}$**	25.9 (10.3)	28.8 (11.8)	21.6 (5.8)	33.5 (10.8)	19.7 (3.5)	28.8 (11.8)	21.6 (5.8)	18.3 (4.8)	27.0 (1.1)	20.8 (1.9)	36.8 (12.9)
$pTau_{181}/A\beta_{1-42}$	0.042 (0.025)	0.059 (0.16)	0.015 (0.005)	0.052 (0.026)	0.033 (0.022)	0.059 (0.016)	0.015 (0.005)	0.12 (0.001)	0.020 (0.005)	0.051 (0.013)	0.068<br>(0.015)

[1] Subset of participants for whom APOE data were available is noted

2.2 Correlation between cortical AD-metaROI measures

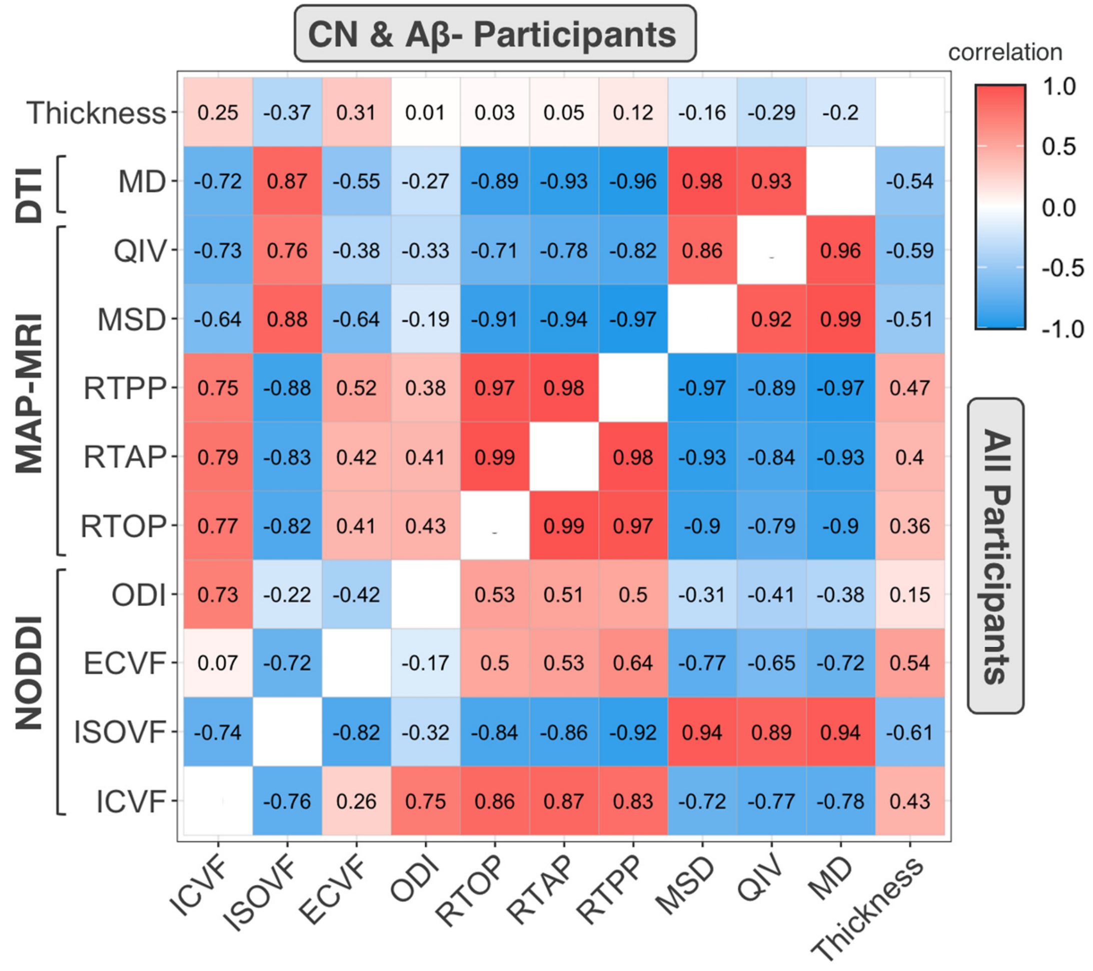

Supplementary Figure 4. Pearson's correlation coefficients between cortical AD-metaROI MRI measures across all participants (N=66; *lower right*) and across the subset of $A\beta_{1-42}$ negative and CU participants (N=29; *upper left*).

2.3 CSF biomarker associations with cortical MRI measures

Supplementary Table 3. Number of significant cortical ROIs —out of 34 individual ROIs and the AD signature meta-ROI tested— and the direction of associations between each cortical MRI and CSF measure (dMRI $p \leq 0.012$; CTh $p \leq 0.0056$).

Model	Measure	Aβ		pTau		pTau/Aβ		Total N
		N	+/-	N	+/-	N	+/-	
MT-NODDI	ECVF			7	-	6	-	13
	ISOVF	15	-	6	+	29	+	50
	ICVF	14	+			20	-	34
	ODI	4	+			4	-	8
MAP-MRI	MSD	6	-			20	+	26
	QIV	16	-	5	+	24	+	45
	RTAP	2	+			9	-	11
	RTOP	1	+			8	-	9
	RTPP	6	+			21	-	27
DTI	MD	13	-			24	+	37
FreeSurfer	CTh	1	+	7	-	5	-	13
Total N		78		25		170		273

2.4 Log10-transformed pTau181 associations

Supplementary Table 4. Summary of log10 transformed pTau results. Number of significant cortical ROIs —out of 34 individual ROIs and the AD signature meta-ROI tested— and the direction of associations between each cortical MRI and CSF measure. When using the log10 transform of pTau instead of pTau, the threshold for significant dMRI associations did not change (dMRI $p \leq 0.012$), while CTh did (CTh $p \leq 0.00086$). Compared to a total of 273 significant associations when assessing Aβ, pTau, and pTau/Aβ, a total of 268 significant associations were detected across Aβ, $\log_{10}$(pTau), and pTau/Aβ.

Model	Measure	Aβ		$\log_{10}$(pTau)		pTau/Aβ		Total N
		N	+/-	N	+/-	N	+/-	
MT-NODDI	ECVF			9	-	6	-	15
	ISOVF	15	-	6	+	29	+	50
	ICVF	14	+			20	-	34
	ODI	4	+			4	-	8
MAP-MRI	MSD	6	-	2	+	20	+	28
	QIV	16	-	1	+	24	+	41
	RTAP	2	+			9	-	11
	RTOP	1	+			8	-	9
	RTPP	6	+			21	-	27
DTI	MD	13	-	1	+	24	+	38
FreeSurfer	CTh			3	-	4	-	7
Total N		77		22		169		268

$\log(pTau_{181})$

Region	ROI	ISOVF (NODDI)	ICVF (NODDI)	ECVF (NODDI)	ODI (NODDI)	QIV (MAP-MRI)	RTPP (MAP-MRI)	RTOP (MAP-MRI)	RTAP (MAP-MRI)	MSD (MAP-MRI)	MD (DTI)	Thickness
	Meta-ROI	0.27 (0.11)	-0.16 (0.13)	-0.27 (0.12)	0 (0.13)	0.23 (0.12)	-0.19 (0.12)	-0.15 (0.13)	-0.16 (0.13)	0.22 (0.12)	0.22 (0.12)	-0.45 (0.12)
frontal	paracentral	0.38 (0.12)	-0.1 (0.13)	-0.42 (0.13)	-0.05 (0.14)	0.32 (0.13)	-0.3 (0.13)	-0.17 (0.13)	-0.17 (0.13)	0.33 (0.13)	0.32 (0.13)	-0.33 (0.14)
frontal	caudalmiddlefrontal	0.34 (0.12)	-0.07 (0.13)	-0.39 (0.12)	0.07 (0.13)	0.27 (0.12)	-0.23 (0.12)	-0.15 (0.13)	-0.15 (0.13)	0.28 (0.12)	0.27 (0.12)	-0.22 (0.14)
frontal	precentral	0.32 (0.12)	-0.1 (0.13)	-0.36 (0.13)	-0.04 (0.13)	0.23 (0.13)	-0.23 (0.13)	-0.16 (0.13)	-0.15 (0.13)	0.27 (0.13)	0.24 (0.13)	-0.32 (0.13)
frontal	superiorfrontal	0.29 (0.12)	0 (0.13)	-0.34 (0.13)	0.06 (0.14)	0.18 (0.12)	-0.17 (0.13)	-0.07 (0.13)	-0.08 (0.13)	0.21 (0.12)	0.2 (0.12)	-0.05 (0.14)
frontal	lateralorbitofrontal	0.23 (0.12)	-0.08 (0.14)	-0.19 (0.13)	0.07 (0.14)	0.16 (0.13)	-0.13 (0.12)	-0.12 (0.13)	-0.15 (0.13)	0.19 (0.12)	0.17 (0.13)	-0.1 (0.14)
frontal	parsorbitalis	0.28 (0.12)	-0.03 (0.14)	-0.28 (0.13)	0.14 (0.14)	0.21 (0.12)	-0.09 (0.13)	0.01 (0.14)	-0.02 (0.13)	0.19 (0.12)	0.19 (0.12)	-0.14 (0.14)
frontal	parstriangularis	0.2 (0.12)	-0.07 (0.13)	-0.24 (0.12)	-0.13 (0.14)	0.15 (0.13)	-0.15 (0.12)	-0.08 (0.12)	-0.07 (0.12)	0.15 (0.12)	0.14 (0.12)	-0.15 (0.13)
frontal	medialorbitofrontal	0.21 (0.12)	-0.19 (0.14)	-0.09 (0.13)	-0.01 (0.14)	0.12 (0.14)	-0.16 (0.13)	-0.17 (0.13)	-0.18 (0.13)	0.17 (0.12)	0.18 (0.13)	-0.02 (0.14)
frontal	rostralmiddlefrontal	0.22 (0.12)	0 (0.13)	-0.3 (0.13)	0.02 (0.14)	0.18 (0.13)	-0.12 (0.13)	-0.05 (0.13)	-0.06 (0.13)	0.17 (0.13)	0.15 (0.13)	-0.04 (0.14)
frontal	frontalpole	0.12 (0.12)	0.13 (0.14)	-0.19 (0.13)	0.19 (0.14)	-0.07 (0.13)	0.01 (0.14)	0 (0.14)	0.02 (0.14)	0.06 (0.13)	0 (0.13)	-0.15 (0.14)
frontal	parsopercularis	0.1 (0.12)	-0.03 (0.13)	-0.13 (0.12)	0.03 (0.14)	0.07 (0.13)	-0.03 (0.12)	0 (0.12)	0.01 (0.12)	0.04 (0.12)	0.04 (0.12)	-0.23 (0.14)
temporal	fusiform	0.26 (0.11)	-0.12 (0.13)	-0.24 (0.12)	0.1 (0.14)	0.23 (0.11)	-0.16 (0.12)	-0.14 (0.13)	-0.17 (0.12)	0.21 (0.12)	0.21 (0.12)	-0.37 (0.14)
temporal	entorhinal	0.21 (0.12)	-0.11 (0.13)	-0.19 (0.13)	-0.21 (0.14)	0.2 (0.13)	-0.22 (0.13)	-0.12 (0.13)	-0.09 (0.13)	0.16 (0.12)	0.19 (0.12)	-0.37 (0.13)
temporal	inferiortemporal	0.23 (0.12)	-0.14 (0.13)	-0.19 (0.13)	0.03 (0.14)	0.19 (0.13)	-0.16 (0.12)	-0.12 (0.13)	-0.14 (0.13)	0.19 (0.12)	0.19 (0.12)	-0.38 (0.13)
temporal	middletemporal	0.24 (0.12)	-0.14 (0.13)	-0.19 (0.12)	0.05 (0.13)	0.2 (0.12)	-0.14 (0.12)	-0.11 (0.13)	-0.13 (0.13)	0.19 (0.12)	0.19 (0.12)	-0.26 (0.13)
temporal	temporalpole	0.28 (0.12)	-0.04 (0.14)	-0.27 (0.12)	0.02 (0.14)	0.24 (0.12)	-0.1 (0.13)	0.08 (0.14)	0.05 (0.14)	0.18 (0.12)	0.23 (0.12)	-0.28 (0.12)
temporal	parahippocampal	0.17 (0.11)	-0.2 (0.13)	-0.07 (0.12)	-0.1 (0.14)	0.2 (0.12)	-0.13 (0.12)	-0.15 (0.13)	-0.15 (0.12)	0.12 (0.12)	0.15 (0.12)	-0.12 (0.14)
temporal	superiortemporal	0.2 (0.12)	-0.07 (0.13)	-0.21 (0.12)	-0.03 (0.13)	0.14 (0.13)	-0.12 (0.13)	-0.05 (0.14)	-0.05 (0.14)	0.14 (0.12)	0.13 (0.12)	-0.24 (0.12)
temporal	bankssts	0.15 (0.12)	-0.07 (0.12)	-0.14 (0.12)	-0.15 (0.14)	0.08 (0.13)	-0.09 (0.12)	-0.04 (0.13)	-0.03 (0.13)	0.09 (0.12)	0.08 (0.12)	-0.25 (0.13)
temporal	transversetemporal	0.11 (0.11)	0 (0.14)	-0.13 (0.12)	-0.02 (0.14)	0.05 (0.12)	-0.03 (0.12)	0.01 (0.13)	0.03 (0.13)	0.04 (0.12)	0.04 (0.12)	0 (0.12)
	insula	0.13 (0.12)	-0.12 (0.13)	-0.11 (0.12)	0.03 (0.13)	0.13 (0.12)	-0.07 (0.12)	-0.03 (0.13)	-0.04 (0.12)	0.08 (0.12)	0.09 (0.12)	-0.16 (0.14)
cingulate	posteriorcingulate	0.29 (0.12)	-0.06 (0.13)	-0.33 (0.12)	0.19 (0.14)	0.22 (0.12)	-0.16 (0.12)	-0.11 (0.13)	-0.13 (0.13)	0.23 (0.12)	0.21 (0.12)	-0.23 (0.14)
cingulate	isthmuscingulate	0.24 (0.12)	-0.14 (0.13)	-0.23 (0.12)	-0.17 (0.13)	0.16 (0.12)	-0.21 (0.12)	-0.13 (0.13)	-0.09 (0.13)	0.18 (0.12)	0.18 (0.12)	-0.34 (0.14)
cingulate	rostralanteriorcingulate	0.05 (0.13)	-0.19 (0.14)	0.02 (0.13)	-0.12 (0.14)	0.05 (0.14)	-0.04 (0.13)	-0.03 (0.13)	0 (0.14)	0 (0.13)	0.02 (0.13)	-0.03 (0.14)
cingulate	caudalanteriorcingulate	0.1 (0.12)	-0.05 (0.14)	-0.09 (0.13)	0.04 (0.14)	0.03 (0.12)	0.01 (0.13)	-0.02 (0.13)	-0.03 (0.14)	0.04 (0.13)	0.03 (0.13)	0.1 (0.14)
parietal	precuneus	0.36 (0.11)	-0.2 (0.13)	-0.39 (0.12)	0.02 (0.13)	0.34 (0.12)	-0.28 (0.12)	-0.21 (0.13)	-0.22 (0.13)	0.32 (0.12)	0.32 (0.12)	-0.43 (0.12)
parietal	superiorparietal	0.34 (0.12)	-0.19 (0.13)	-0.35 (0.13)	-0.12 (0.13)	0.32 (0.13)	-0.28 (0.13)	-0.18 (0.14)	-0.18 (0.14)	0.31 (0.13)	0.3 (0.13)	-0.46 (0.12)
parietal	postcentral	0.36 (0.12)	-0.13 (0.12)	-0.4 (0.12)	-0.18 (0.12)	0.29 (0.12)	-0.3 (0.12)	-0.18 (0.13)	-0.17 (0.13)	0.32 (0.12)	0.3 (0.12)	-0.28 (0.13)
parietal	inferiorparietal	0.2 (0.12)	-0.11 (0.13)	-0.23 (0.13)	-0.15 (0.13)	0.15 (0.13)	-0.14 (0.13)	-0.08 (0.13)	-0.07 (0.13)	0.14 (0.13)	0.13 (0.13)	-0.31 (0.13)
parietal	supramarginal	0.16 (0.12)	-0.04 (0.13)	-0.22 (0.13)	-0.08 (0.13)	0.09 (0.12)	-0.1 (0.12)	-0.04 (0.13)	-0.03 (0.13)	0.1 (0.13)	0.09 (0.12)	-0.32 (0.13)
occipital	lateraloccipital	0.25 (0.12)	-0.14 (0.12)	-0.21 (0.14)	0.02 (0.14)	0.21 (0.12)	-0.15 (0.12)	-0.13 (0.13)	-0.14 (0.12)	0.19 (0.12)	0.18 (0.12)	-0.35 (0.13)
occipital	pericalcarine	0.22 (0.12)	-0.12 (0.13)	-0.23 (0.13)	-0.06 (0.14)	0.18 (0.12)	-0.14 (0.13)	-0.09 (0.13)	-0.1 (0.13)	0.17 (0.13)	0.17 (0.12)	0.07 (0.14)
occipital	cuneus	0.25 (0.11)	0.06 (0.13)	-0.37 (0.12)	-0.05 (0.13)	0.15 (0.12)	-0.12 (0.13)	0.01 (0.14)	0.02 (0.13)	0.15 (0.12)	0.14 (0.12)	-0.03 (0.14)
occipital	lingual	0.17 (0.12)	-0.11 (0.12)	-0.15 (0.13)	-0.06 (0.13)	0.14 (0.11)	-0.09 (0.13)	-0.06 (0.13)	-0.07 (0.13)	0.11 (0.12)	0.12 (0.12)	-0.08 (0.14)

Absolute Standardized *Beta*: 0.60 – 0.23

Supplementary Figure 5. Effect sizes (*Beta*-values and standard error) for associations between log10-transformed CSF $pTau_{181}$ and each cortical MRI measure. Significant associations (dMRI $p \leq 0.012$; CTh $p \leq 0.00086$) are shaded according to the absolute value of their effect size. For comparison with raw $pTau_{181}$, ROIs that changed significance are highlighted in red boxes.

2.5 Sensitivity analysis: CSF biomarker associations in non-demented participants

Supplementary Table 5. Number of significant ROIs (dMRI $p \leq 0.048$; CTh $p \leq 0.029$) and direction of associations between each cortical MRI and CSF measure in the subset of participants without dementia (N=64). Only MRI measures and ROIs that were significant in the entire cohort were evaluated. The percent of the ROIs significant in the non-demented subset relative to the whole cohort is reported. Only four of 170 p-tau$_{181}$/A$\beta_{1\text{–}42}$ ROIs that were significant in the full cohort were not significant in non-demented participants.

**Model**	**Measure**	**A$\beta_{1\text{–}42}$**			**p-tau$_{181}$**			**p-tau$_{181}$/A$\beta_{1\text{–}42}$**			**Total N (%)**
		**N**	**%**	**+/-**	**N**	**%**	**+/-**	**N**	**%**	**+/-**	
MT-NODDI	ECVF				7	100	-	6	100	-	13 (100%)
	ISOVF	15	100	-	6	100	+	29	100	-	50 (100%)
	ICVF	14	100	+				20	100	-	34 (100%)
	ODI	4	100	+				4	100	-	8 (100%)
MAPMRI	MSD	6	100	-				20	100	+	26 (100%)
	QIV	16	100	-	5	100	+	22	91.7	+	43 (95.6%)
	RTAP	2	100	+				9	100	-	11 (100%)
	RTOP	1	100	+				8	100	-	9 (100%)
	RTPP	6	100	+				20	95.2	-	26 (96.3%)
DTI	MD	13	100	-				23	95.8	+	36 (97.3%)
FreeSurfer	CTh	1	100	+	7	100	-	5	100	-	13 (100%)
Total N (%)		78 (100%)			25 (100%)			166 (97.6%)			269 (98.5%)

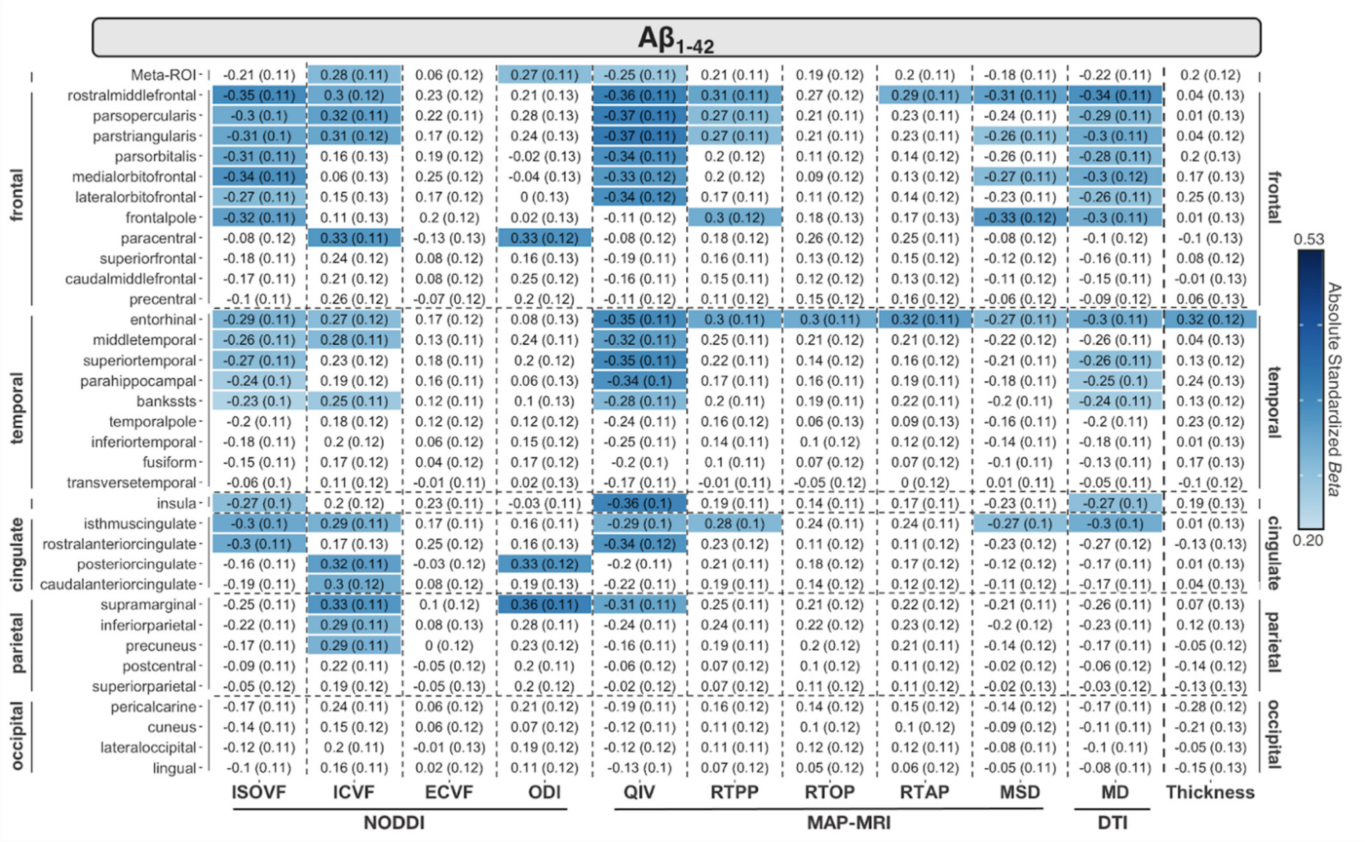

Supplementary Figure 6. Effect sizes (*Beta*-values and standard error) for associations between CSF Aβ$_{1–42}$ and each cortical MRI measure in the subset of participants without dementia (N=64). Significant associations (dMRI $p \leq 0.048$; CTh $p \leq 0.029$) are shaded according to the absolute value of their effect size; only measures and ROIs that were significant in the entire cohort were considered. All ROIs that were significant in the full cohort remained significant in non-demented participants.

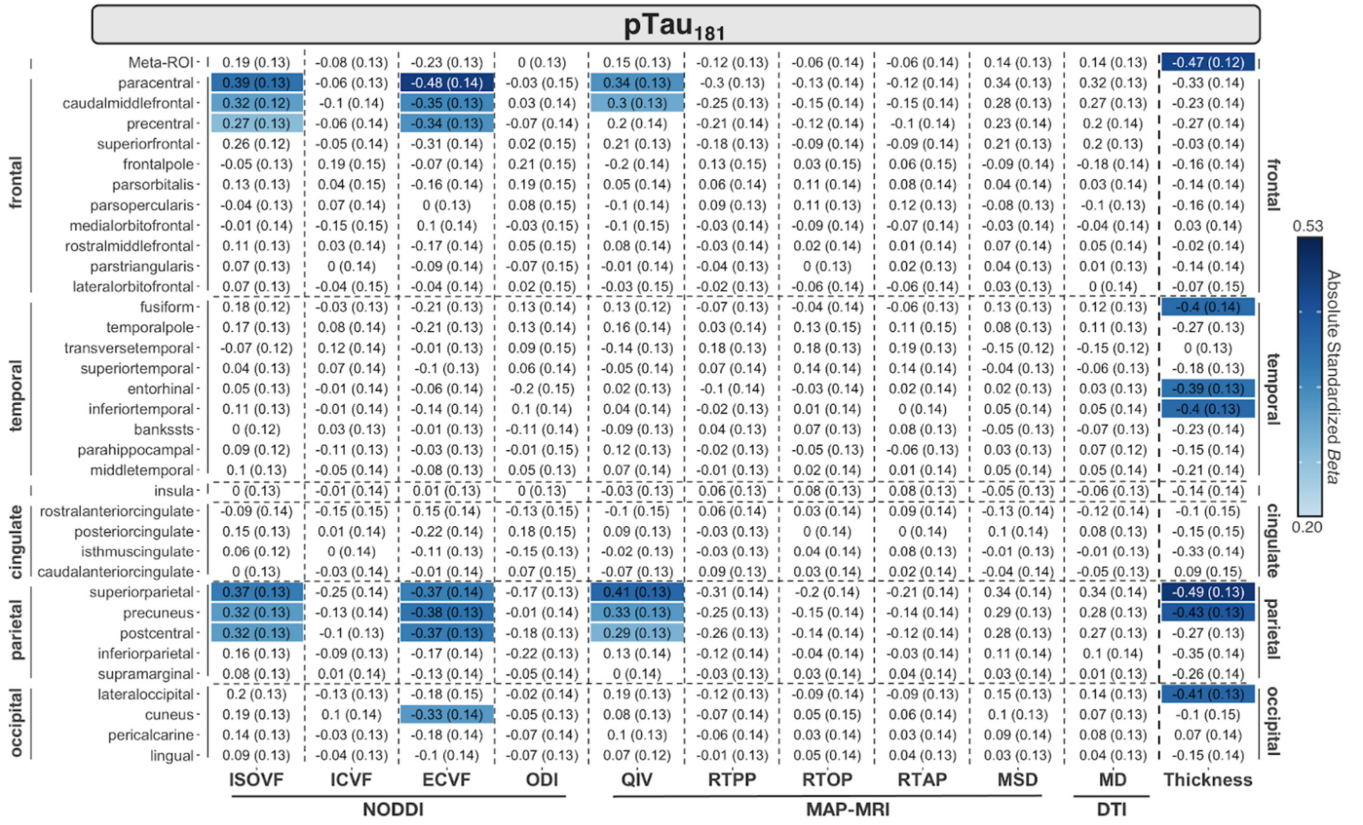

Supplementary Figure 7. Effect sizes (*Beta*-values and standard error) for associations between CSF $pTau_{181}$ and each cortical MRI measure in the subset of participants without dementia (N=64). Significant associations (dMRI $p \leq 0.048$; CTh $p \leq 0.029$) are shaded according to the absolute value of their effect size; only measures and ROIs that were significant in the entire cohort were considered. All ROIs that were significant in the full cohort remained significant in non-demented participants.

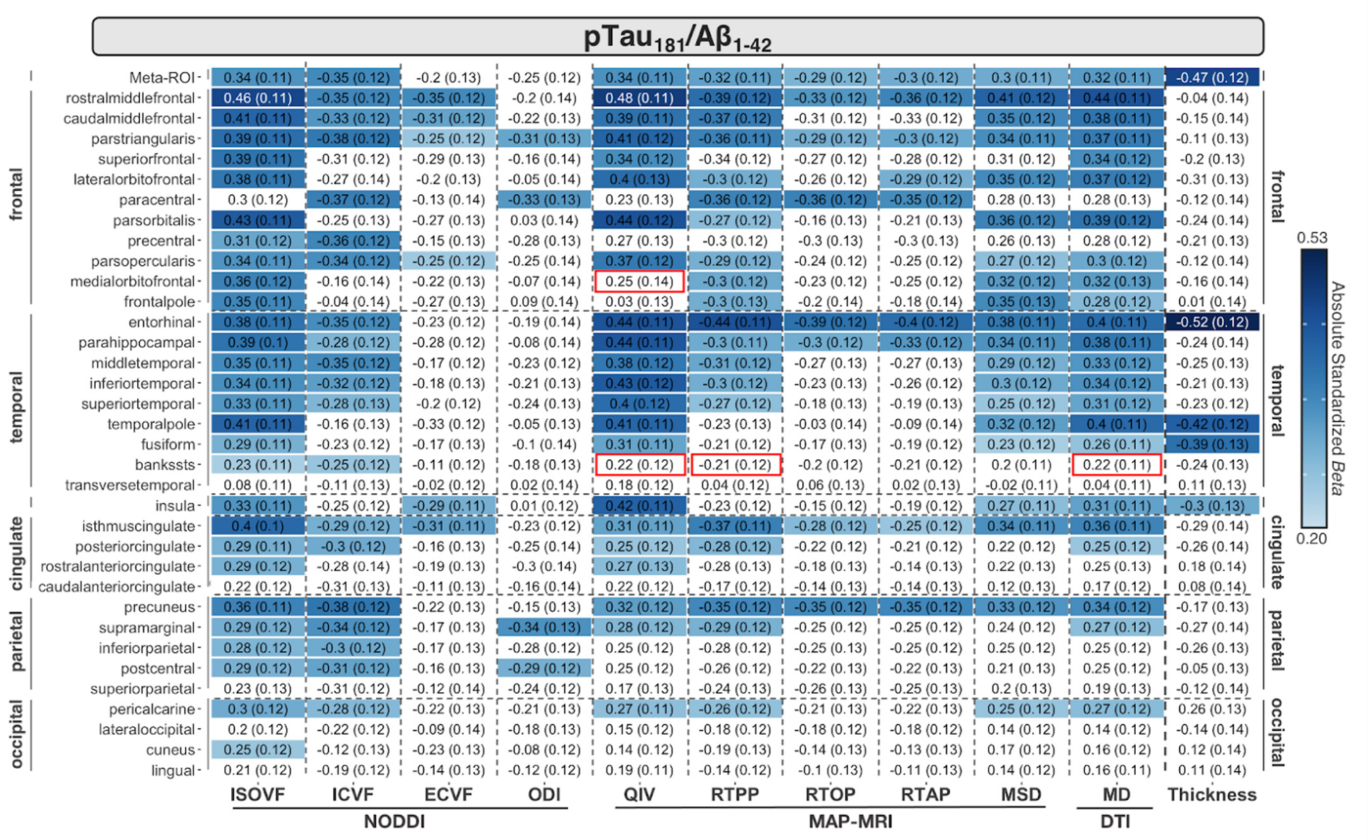

Supplementary Figure 8. Effect sizes (*Beta*-values and standard error) for associations between CSF $pTau_{181}/A\beta_{1–42}$ and each cortical MRI measure in the subset of participants without dementia (N=64). Significant associations (dMRI $p \leq 0.048$; CTh $p \leq 0.029$) are shaded according to the absolute value of their effect size; only measures and ROIs that were significant in the entire cohort were considered. Only four ROIs that were significant in the full cohort were not significant in non-demented participants; these are highlighted in red boxes.

2.6 Clinical diagnosis differences in cortical MRI measures

Supplementary Table 6. Number of significant cortical ROIs —out of 34 individual ROIs and the AD signature meta-ROI tested— and the direction of associations between each cortical MRI and clinical diagnosis, i.e., cognitively impaired (CU) vs cognitively impaired (CI) (dMRI $p \leq 0.019$; CTh $p \leq 0.0076$). Note: a positive association suggests measures were higher in CI than CU.

Model	Measure	CU v CI	
		**N**	**+/-**
MT-NODDI	ECVF	8	-
	ISOVF	26	+
	ICVF	17	-
	ODI	3	-
MAP-MRI	MSD	15	+
	QIV	23	+
	RTAP	8	-
	RTOP	6	-
	RTPP	14	-
DTI	MD	18	+
FreeSurfer	CTh	7	-
Total N		145	

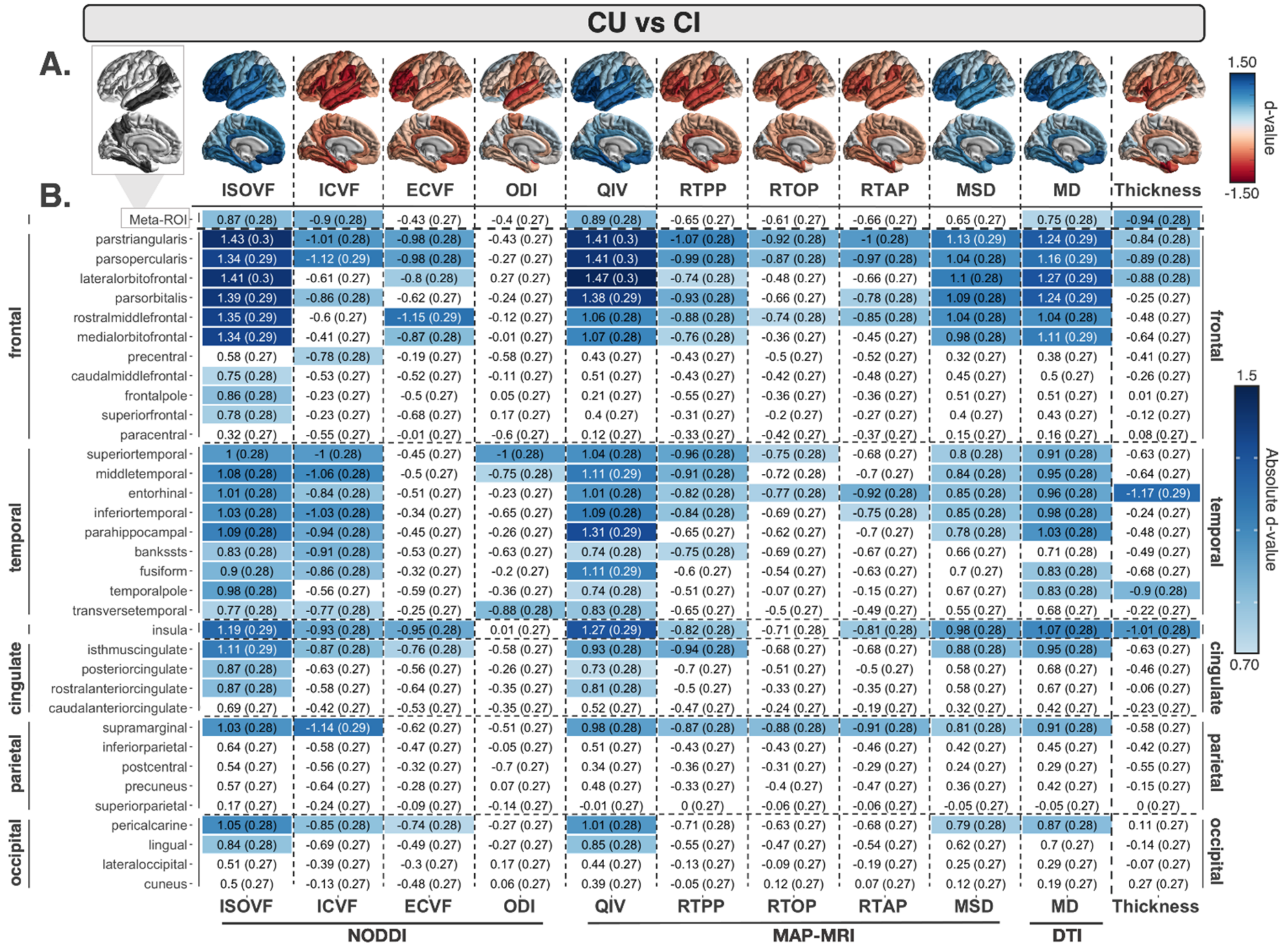

Supplementary Figure 9. Effect sizes (d-values and standard error) for differences in each cortical measure between cognitively unimpaired (CU) and cognitively impaired (CI defined as MCI or dementia) participants. Significant associations (dMRI $p \leq 0.019$; CTh $p \leq 0.0076$) are shaded according to the absolute value of their effect size. Effect sizes were estimated by converting t-values from random effects multiple linear regressions to d statistics: $d = t(n1+n2)\ /\ \sqrt{n1n2} * \sqrt{df}$, where $n1$ and $n2$ are the sample size for each group. As in previous studies that evaluated clinical differences in cortical NODDI ICVF and ODI, we found reduced ICVF and ODI in cognitively impaired participants (10, 11). Like the differences between MCI and CU participants reported in Vogt et al. (11), we detected greater ICVF differences compared to ODI and CTh, particularly in the temporal lobe. Significant cortical DTI MD effect sizes were comparable to those reported in a meta-analyses of cortical, hippocampal, and white matter MD difference between CU and MCI (Hedges' g=0.32-1.08)(12). An effect size of d=1.08 requires a sample size of 15 cases and 15 controls to detect with 80% power; our sample size exceeds the required sample size needed to detect this effect.

2.7 AD-metaROI MRI differences between CSF and clinical diagnosis group

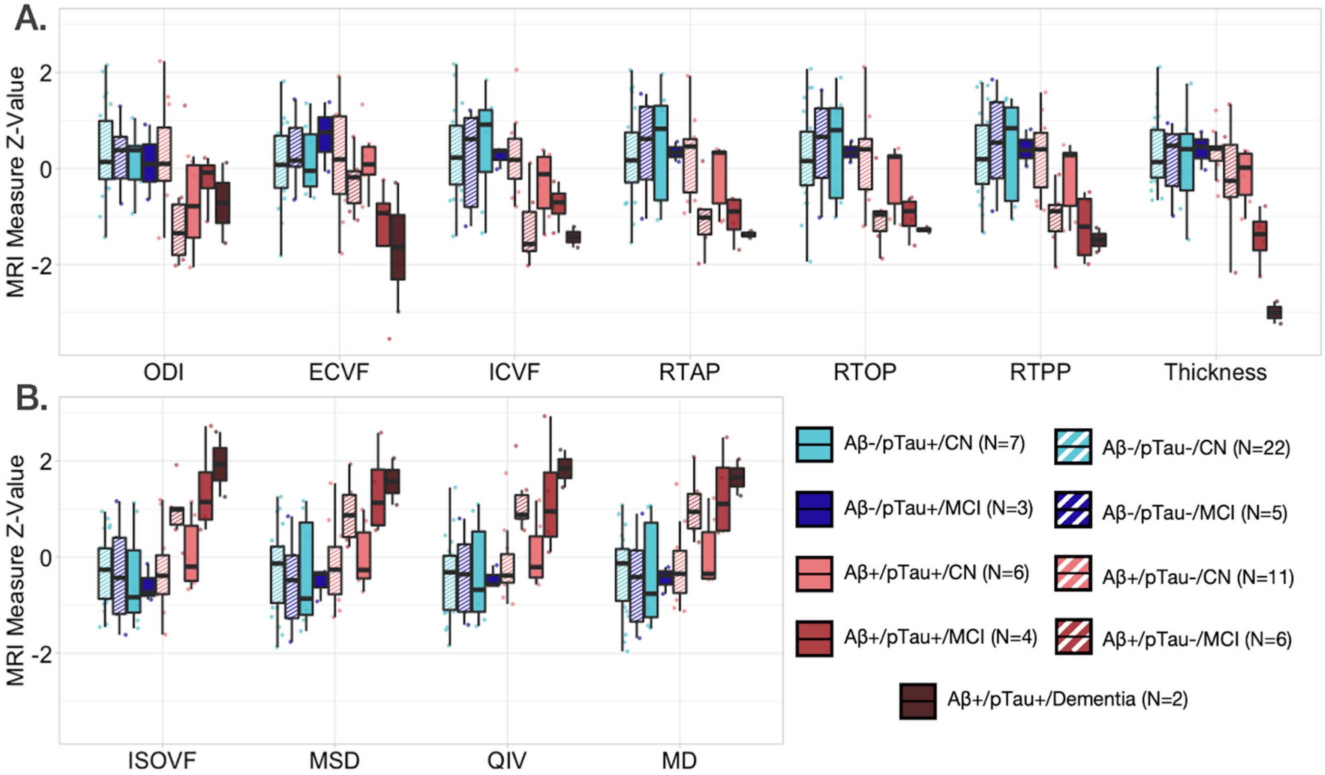

Supplementary Figure 10. Scaled cortical AD-metaROI MRI measures classified based on four CSF biomarker cutoff subgroups and three clinical diagnoses show **(A)** lower MT-NODDI ODI, ECVF, ICVF, MAPMRI RTAP, RTOP, RTPP, and FreeSurfer CTh (*top panel*) and **(B)** higher MT-NODDI ISOVF, MAPMRI MSD, QIV, and DTI MD (*bottom panel*) in Aβ+ (*reds*) compared to Aβ- (*blues*) individuals. While group sizes are very small (as low as 2 to 6 participants), the greatest metaROI differences are visible in Aβ+ participants with either MCI or Dementia, regardless of pTau status (pTau- are depicted in *stripes* while pTau+ are *solid*).

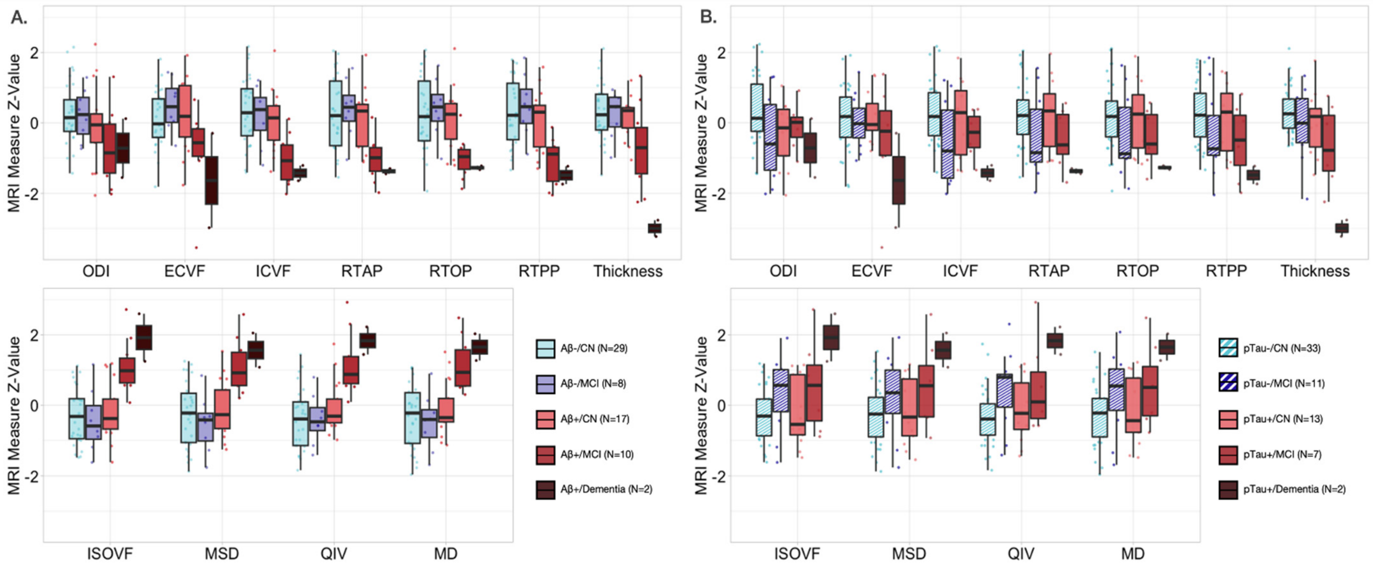

Supplementary Figure 11. Scaled cortical AD-metaROI measures classified based on **(A)** Aβ+/- cutoff subgroups (*reds* vs *blues*) show the greatest dMRI differences in Aβ+ participants with either MCI or dementia. **(B)** Scaled metaROI measures classified based on pTau +/- cutoff subgroups (*solid* vs *striped*) only show large dMRI differences in the two pTau+ participants with dementia, while MCI participants show moderate differences regardless of pTau status.

2.8 Interactive effects of CSF biomarker on AD-metaROI MRI measures

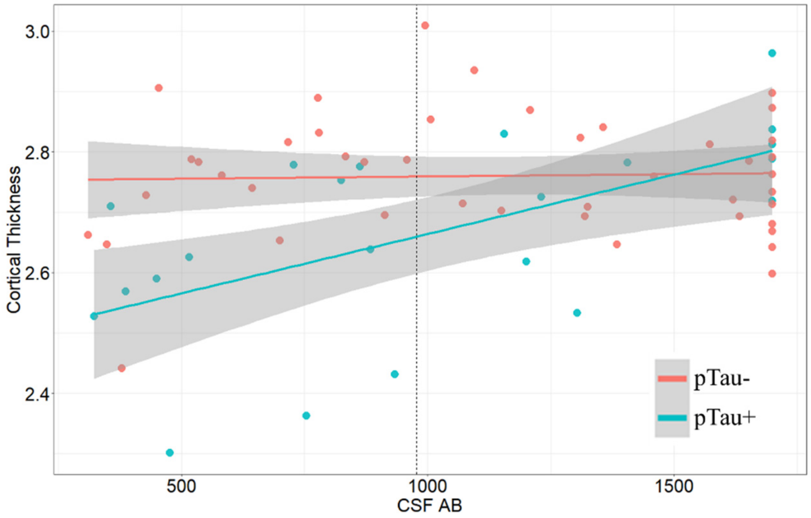

Supplementary Figure 12. The relationship between AD-metaROI cortical thickness and CSF Aβ concentration was significantly moderated by pTau group (pTau+ vs -). The dotted line on the x-axis indicates the CSF Aβ cut-point for positivity.

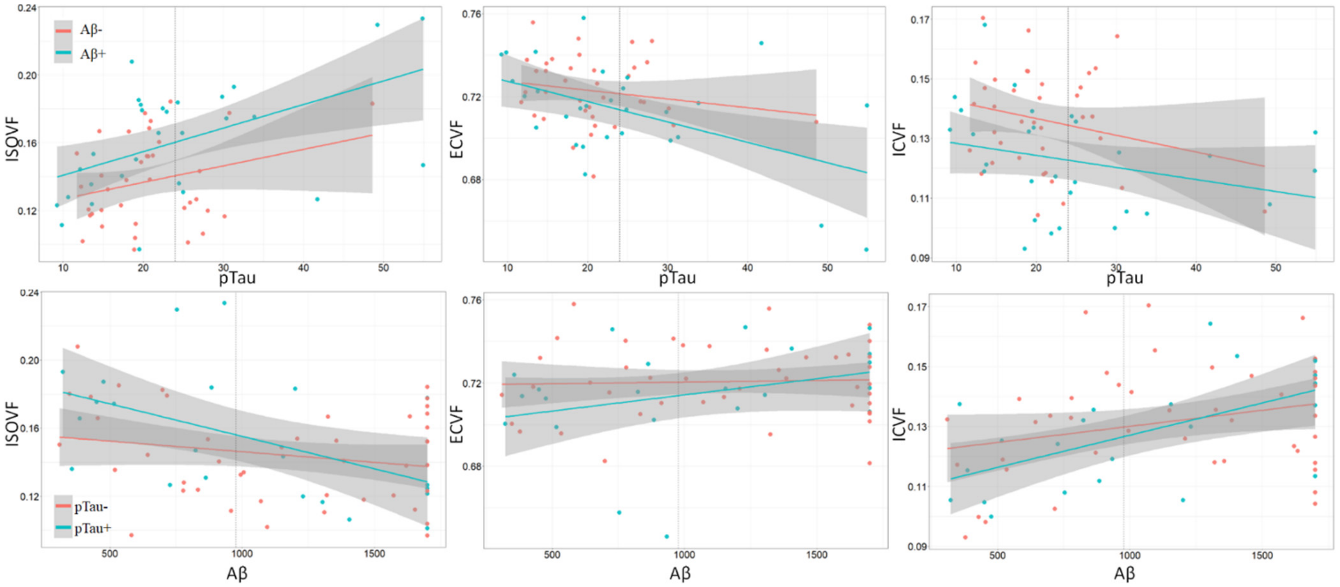

Supplementary Figure 13. While no significant interactive effects were detected, the relationship between AD-metaROI NODDI measures and CSF pTau concentration in either Aβ+ or Aβ- participants (*top row*) or CSF Aβ concentration in either pTau+ or pTau- participants (*bottom row*) are shown for reference. Dotted lines on the x-axis indicate the CSF biomarker cut-points for each measure.

2.9 Exploratory CSF biomarker group classification by cortical AD-metaROI measures

2.9.1 Non-residualized cortical AD-metaROI measure CSF group classification

2.9.1.1 Logistic regression classification across participants

Supplementary Table 7. For each cortical AD-metaROI measure (not residualized), dichotomized CSF biomarker classification area under the curve (AUC).

Model	Measure	Full Cohort (N=66)			Exclude Dementia (N=64)		
		**Aβ**	**pTau**	**pTau/Aβ**	**Aβ**	**pTau**	**pTau/Aβ**
**MT-NODDI**	**ECVF**	0.71	0.55	0.76**	0.71	0.53	0.75*
	**ICVF**	0.78**	0.57	0.88**	0.78**	0.53	0.86**
	**ISOVF**	0.76**	0.57	0.87**	0.75**	0.48	0.86**
	**ODI**	0.74**	0.59	0.76**	0.74**	0.57	0.75**
**MAP-MRI**	**RTOP**	0.76**	0.58	0.84**	0.76**	0.46	0.82**
	**RTAP**	0.76**	0.57	0.85**	0.75**	0.47	0.84**
	**RTPP**	0.77**	0.59	0.86**	0.76**	0.45	0.85**
	**MSD**	0.77**	0.57	0.86**	0.76**	0.46	0.85**
	**QIV**	**0.82****	0.58	**0.92****	**0.80****	0.54	**0.91****
**DTI**	**MD**	0.79**	0.58	0.88**	0.78**	0.46	0.88**
**FreeSurfer**	**Thickness**	0.73**	**0.67****	0.79**	0.72*	**0.64**	0.77**

**Full cohort: dMRI $p \leq 0.021$, CTh $p \leq 0.016$
**No Dementia: dMRI $p \leq 0.029$, CTh $p \leq 0.0036$
*p<0.05

2.9.1.2 Five-fold logistic regression classification 80-20 split

As reported in **Supplementary Figure 14** and **Supplementary Table 8**, dMRI AD-metaROI measures better distinguished Aβ+ from Aβ- participants than did cortical thickness (mean AUC=0.66); ISOVF, RTPP, MSD, and MD performed similarly (AUC=0.75-0.77) and QIV performed marginally better (AUC=0.79). In contrast, for $pTau_{181}$ classification, cortical thickness (AUC=0.69) and dMRI ISOVF, RTOP, RTAP, RTPP, QIV and MD (AUC=0.65-0.66) performed marginally better than ECVF, ICVF, and ODI. $pTau_{181}/A\beta_{1\text{–}42}$ status was also better classified by dMRI measures (AUC=0.87-0.89) than by cortical thickness (AUC=0.76), excluding ECVF and ODI. Similar classification trends were found in the subset of participants without dementia, the most notable differences being that compared to the full group 1) measures of restriction and ODI better classified Aβ status, and 2) there was an even more notable difference in $pTau_{181}$ classification accuracy between diffusion and thickness measures, with diffusion measures performing on average marginally worse than in the full group and cortical thickness performing better (AUC=0.76).

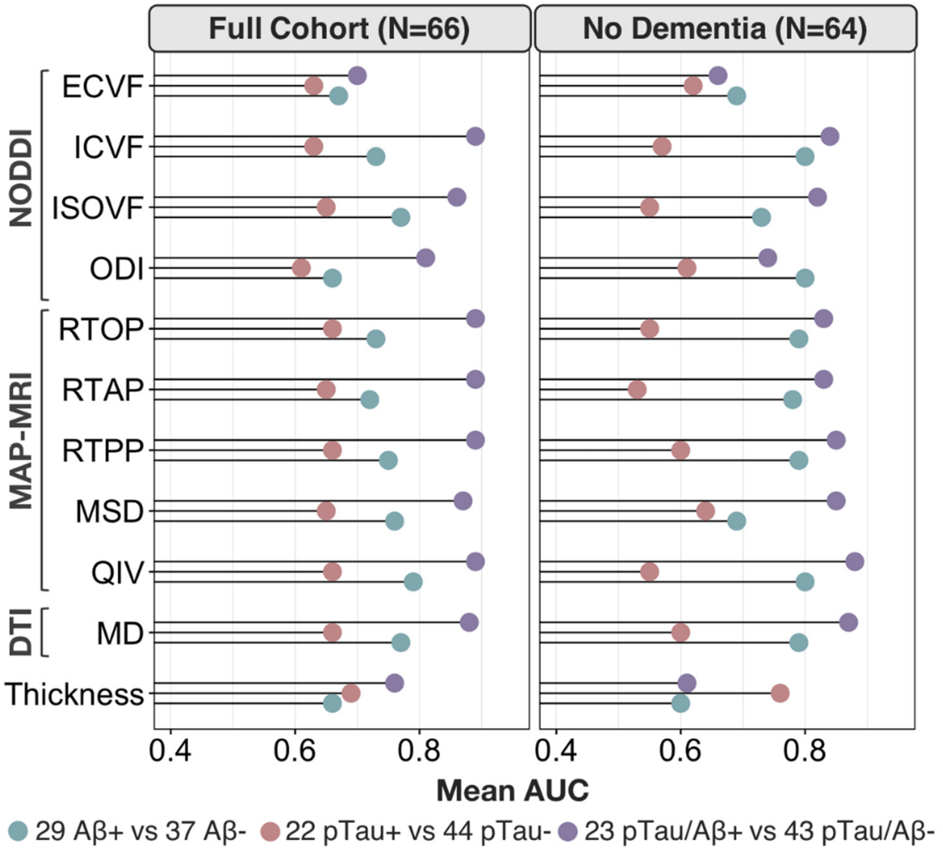

Supplementary Figure 14. For each cortical AD-metaROI measure, the average dichotomized CSF biomarker group classification area under the curve (AUC) from 5 folds is reported.

Supplementary Table 8. For each cortical AD-metaROI measure (not residualized), the average dichotomized CSF biomarker classification area under the curve (AUC) from 5 folds is reported.

Model	Measure	Full Cohort (N=66)			Exclude Dementia (N=64)		
		Aβ	pTau	pTau/Aβ	Aβ	pTau	pTau/Aβ
MT-NODDI	ECVF	0.67	0.63	0.70	0.69	0.62	0.66
	ICVF	0.73	0.63	**0.89**	**0.80**	0.57	0.84
	ISOVF	0.77	0.65	0.86	0.73	0.55	0.82
	ODI	0.66	0.61	0.81	**0.80**	0.61	0.74
MAP-MRI	RTOP	0.73	0.66	**0.89**	0.79	0.55	0.83
	RTAP	0.72	0.65	**0.89**	0.78	0.53	0.83
	RTPP	0.75	0.66	**0.89**	0.79	0.60	0.85
	MSD	0.76	0.65	0.87	0.69	0.64	0.85
	QIV	**0.79**	0.66	**0.89**	**0.80**	0.55	**0.88**
DTI	MD	0.77	0.66	0.88	0.79	0.60	0.87
FreeSurfer	CTh	0.66	**0.69**	0.76	0.60	**0.76**	0.61

2.9.2 Residualized cortical AD-metaROI measure CSF group classification

2.9.2.1 Logistic regression classification across participants

As reported in **Supplementary Figure 15** and **Supplementary Table 9**, residualized dMRI AD-metaROI measures (i.e., adjusted for age, sex, and education) significantly distinguished Aβ+ from Aβ- participants with similar performance from ICVF, ISOVF, RTPP, MSD, and MD (AUC=0.77-0.79), and QIV performing marginally better (AUC=0.82). In contrast, only cortical thickness significantly classified $pTau_{181}$ (AUC=0.70). $pTau_{181}/A\beta_{1\text{–}42}$ status was also best classified by ICVF (AUC=0.81), MD (AUC=0.82), and QIV (AUC=0.85). Similar classification trends were found in the subset of participants without dementia.

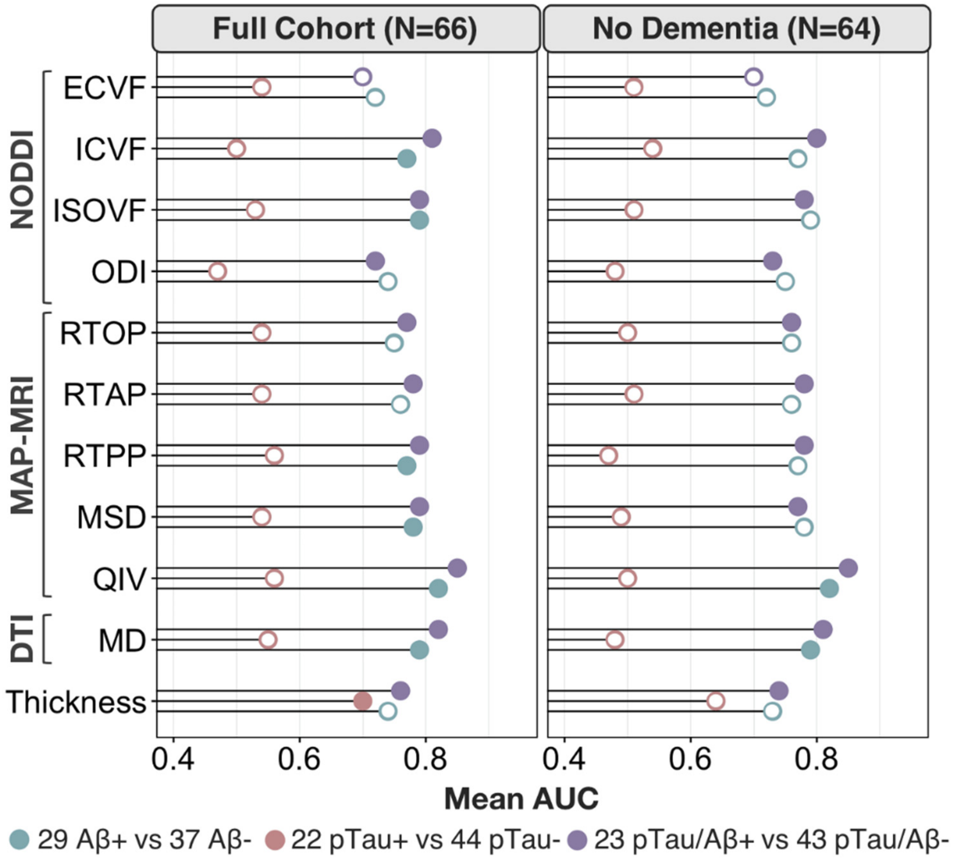

Supplementary Figure 15. For each residualized cortical AD-metaROI measure, the area under the curve (AUC) of the dichotomized CSF group classifications are reported. Significant associations after multiple comparisons correction are indicated by filled circles (Full cohort: dMRI $p \leq 0.020$, CTh $p \leq 0.035$; No Dementia: dMRI $p \leq 0.017$, CTh $p \leq 0.17$).

Supplementary Table 9. For each residualized cortical AD-metaROI measure, dichotomized CSF biomarker classification area under the curve (AUC) is reported.

Model	Measure	Full Cohort (N=66)			Exclude Dementia (N=64)		
		**Aβ**	**pTau**	**pTau/Aβ**	**Aβ**	**pTau**	**pTau/Aβ**
**MT-NODDI**	**ECVF**	0.72	0.54	0.70	0.72	0.51	0.70
	**ICVF**	0.77**	0.50	0.81**	0.77*	0.54	0.80**
	**ISOVF**	0.79**	0.53	0.79**	0.79*	0.51	0.78**
	**ODI**	0.74*	0.47	0.72**	0.75*	0.48	0.73**
**MAP-MRI**	**RTOP**	0.75*	0.54	0.77**	0.76	0.50	0.76**
	**RTAP**	0.76*	0.54	0.78**	0.76*	0.51	0.78**
	**RTPP**	0.77**	0.56	0.79**	0.77*	0.47	0.78**
	**MSD**	0.78**	0.54	0.79**	0.78*	0.49	0.77**
	**QIV**	**0.82****	0.56	**0.85****	**0.82****	0.50	**0.85****
**DTI**	**MD**	0.79**	0.55	0.82**	0.79**	0.48	0.81**
**FreeSurfer**	**CTh**	0.74**	**0.70****	0.76**	0.73	**0.64**	0.74**

** Full cohort: dMRI $p \leq 0.020$, CTh $p \leq 0.035$
**No Dementia: dMRI $p \leq 0.017$, CTh $p \leq 0.17$
* p<0.05

2.9.2.2 Five-fold logistic regression classification 80-20 split

As reported in **Supplementary Figure 16** and **Supplementary Table 10**, residualized dMRI QIV AD-metaROI measures best distinguished Aβ+ from Aβ- participants (mean AUC=0.80). In contrast, for $pTau_{181}$ classification cortical thickness (AUC=0.72), RTAP, and RTOP (AUC=0.70-0.71) performed marginally better than other MRI measures. $pTau_{181}/A\beta_{1\text{-}42}$ status was best classified by dMRI measures (AUC=0.82-0.85), excluding ECVF, with ICVF and QIV performing marginally better (AUC=0.85). Similar classification trends were found in the subset of participants without dementia, the most notable differences being that compared to the full group 1) measures of restriction and ODI better classified Aβ status, and 2) there was an even more notable difference in $pTau_{181}$ classification accuracy between diffusion and thickness measures, with diffusion measures performing on average marginally worse than in the full group and cortical thickness performing better (AUC=0.76).

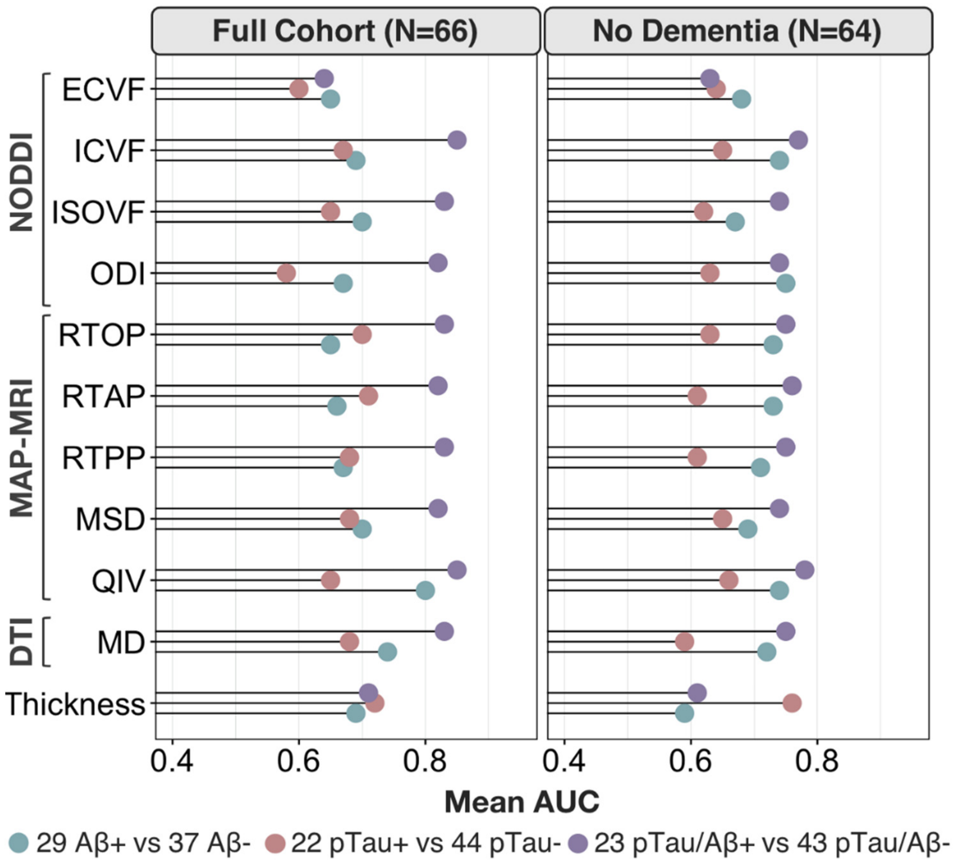

Supplementary Figure 16. For each residualized cortical AD-metaROI MRI measure, the average dichotomized CSF biomarker classification area under the curve (AUC) from 5 folds is reported.

Supplementary Table 10. For each residualized cortical AD-metaROI measure, the average dichotomized CSF biomarker classification area under the curve (AUC) from 5 folds is reported.

Model	Measure	Full Cohort (N=66)			Exclude Dementia (N=64)		
		Aβ	pTau	pTau/Aβ	Aβ	pTau	pTau/Aβ
MT-NODDI	ECVF	0.65	0.60	0.64	0.68	0.64	0.63
	ICVF	0.69	0.67	**0.85**	0.74	0.65	0.77
	ISOVF	0.70	0.65	0.83	0.67	0.62	0.74
	ODI	0.67	0.58	0.82	**0.75**	0.63	0.74
MAP-MRI	RTOP	0.65	0.70	0.83	0.73	0.63	0.75
	RTAP	0.66	0.71	0.82	0.73	0.61	0.76
	RTPP	0.67	0.68	0.83	0.71	0.61	0.75
	MSD	0.70	0.68	0.82	0.69	0.65	0.74
	QIV	**0.80**	0.65	**0.85**	0.74	0.66	**0.78**
DTI	MD	0.74	0.68	0.83	0.72	0.59	0.75
FreeSurfer	CTh	0.69	**0.72**	0.71	0.59	**0.76**	0.61

2.10 Sensitivity analysis: Mediation analyses in non-demented participants

We conducted sensitivity analyses to determine whether the 10 significant mediating effects of cortical MRI measures on the relationship between CSF biomarkers and delayed memory would hold after excluding individuals with dementia (N=2). Overall, mediation results were similar to those in the whole sample **(Supplementary Table 11**). Specifically, lower A$\beta_{1\text{-}42}$ (p=0.02; β=0.27) and greater pTau$_{181}$/A$\beta_{1\text{-}42}$ (p=5.6x10^{-4}; β =-0.43) were associated with poorer delayed memory performance. As in the full group, the effect of A$\beta_{1\text{-}42}$ on delayed memory was fully mediated by ICVF and QIV, while the effect of pTau$_{181}$/A$\beta_{1\text{-}42}$ was partially mediated by all eight dMRI AD-metaROI measures tested.

Supplementary Table 11. Sensitivity mediation analyses in participants without dementia.

**Memory (DV)**	**CSF (IV)**	**MRI Model**	**Cortical Mediator**	**Direct Effect**		**Mediation Effect**		
				**β**	**P**	**β**	**P**	**%**
**WMS Logical Memory** (N=64)	**Aβ** p=0.023 β=0.27	MT-NODDI	ICVF	0.19	0.098	0.083	**0.034****	30.1
		MAP-MRI	QIV	0.17	0.16	0.097	**0.016****	36.2
	**pTau/Aβ** p=5.6x10^{-4} β=-0.43	MT-NODDI	ICVF	-0.35	**0.004****	-0.09	**0.048****	20.2
			ISOVF	-0.33	**0.008****	-0.10	**0.026****	23.8
		MAP-MRI	MSD	-0.34	**0.006****	-0.09	**0.026****	24.6
			QIV	-0.30	**0.012****	-0.10	**0.028****	24.8
			RTAP	-0.35	**0.004****	-0.09	**0.028****	25.2
			RTOP	-0.36	**0.002****	-0.08	**0.044****	23.7
			RTPP	-0.34	**0.006****	-0.09	**0.038****	22.6
		DTI	MD	-0.33	**0.01****	-0.10	**0.022****	24.4

**Indirect p < FDR Threshold for 10 tests; Direct $p \leq 0.012$; Mediation $p \leq 0.048$

2.11 ADSP-PHC cognitive domain mediation analyses

Supplementary Table 12. Mean ADSP-PHC cognitive composite scores for study participants.

	Total	CSF Aβ or pTau Group				CSF pTau/Aβ Ratio Group		CSF Aβ and pTau Group			
		**Aβ+**	**Aβ-**	**pTau+**	**pTau-**	**pTau/Aβ +**	**pTau/Aβ -**	**Aβ-/pTau-**	**Aβ-/pTau+**	**Aβ+/pTau-**	**Aβ+/pTau+**
**N**	66	29	37	22	44	23	43	27	10	17	12
**Memory (SD)**	0.56 (0.66)	0.40 (0.79)	0.69 (0.50)	0.30 (0.73)	0.69 (0.58)	0.22 (0.76)	0.74 (0.52)	0.75 (0.49)	0.53 (0.52)	0.60 (0.71)	0.11 (0.85)
**Executive Function[1] (SD)**	0.66 (0.66)<br>N=64	0.58 (0.61)<br>N=28	0.87 (0.61)<br>N=36	0.46 (0.67)<br>N=21	0.75 (0.63)<br>N=43	0.26 (0.63)<br>N=22	0.87 (0.57)<br>N=42	0.88 (0.62)	0.86 (0.62)<br>N=9	0.53 (0.62)<br>N=16	0.17 (0.55)
**Language[1] (SD)**	0.75 (0.62)<br>N=65	0.61 (0.62)<br>N=28	0.86 (0.61)	0.54 (0.50)	0.86 (0.65)<br>N=43	0.47 (0.64)<br>N=22	0.90 (0.56)	0.90 (0.66)	0.75 (0.46)	0.80 (0.66)<br>N=16	0.37 (0.49)
**Visuospatial Function[1] (SD)**	0.09 (0.32)<br>N=29	-0.04 (0.42)<br>N=12	0.18 (0.18)<br>N=17	0.03 (0.38)<br>N=12	0.13 (0.27)<br>N=17	-0.11 (0.44)<br>N=10	0.19 (0.17)<br>N=19	0.18 (0.17)<br>N=11	0.18 (0.20)<br>N=6	0.012 (0.38)<br>N=6	-0.11 (0.48)<br>N=6

[1] Subset of total participants for which data was available is noted.

2.11.1 AD-metaROI MRI Mediators Evaluated

By convention, mediation analyses were performed only for AD-metaROI cortical measures that were significantly associated with CSF biomarkers. In our primary analyses, CSF $A\beta_{1–42}$ was significantly associated with QIV, ICVF, and ODI AD-metaROI measures (**Figure 1**), $pTau_{181}$ was associated with AD-metaROI CTh (**Figure 2**), and $pTau_{181}/A\beta_{1–42}$ with CTh and all AD-metaROI measures except ODI and ECVF (**Figure 3**).

2.11.2 ADSP-PHC Composite Memory

Lower CSF $A\beta_{1–42}$ was associated with poorer memory performance (p=0.03; β=0.23; N=66), as hypothesized. The effect of $A\beta_{1–42}$ on memory was fully mediated by QIV (41.7% of the total effect; **Supplementary Table 13**).

While we did not find significant associations between raw CSF $pTau_{181}$ values and memory (p=0.10), log_{10}-transformed $pTau_{181}$ was associated with memory (p=0.03; β =-0.27), and CTh fully mediated this effect (64.1% of the total effect; **Supplementary Table 13**).

Greater $pTau_{181}/A\beta_{1–42}$ was also associated with poorer memory performance (p=0.0015; β =-0.37). ISOVF fully mediated the effect of $pTau_{181}/A\beta_{1–42}$ on memory (36.9% of the total effect; **Supplementary Table 13**).

2.11.3 ADSP-PHC Composite Executive Function

Lower CSF $A\beta_{1–42}$ was associated with poorer executive function (p=0.005; β=0.32; N=64) as was greater $pTau_{181}/A\beta_{1–42}$ (p=0.03; β =-0.27), but no MRI measures were found to mediate these effects.

We did not find significant associations between CSF $pTau_{181}$ (p=0.69) or log_{10}-transformed $pTau_{181}$ (p=0.47) and executive function.

2.11.4 ADSP-PHC Composite Language

We did not find significant associations between CSF $A\beta_{1–42}$ (p=0.15), $pTau_{181}$ (p=0.39), or log_{10}-transformed $pTau_{181}$ (p=0.28) and language (N=65). Greater $pTau_{181}/A\beta_{1–42}$ was associated with worse language (p=0.02; β =-0.28), but no MRI measures were found to mediate this effect.

2.11.5 ADSP-PHC Composite Visuospatial Functioning

We did not find significant associations between CSF $A\beta_{1–42}$ (p=0.20), $pTau_{181}$ (p=0.83), log_{10}-transformed $pTau_{181}$ (p=0.85), or $pTau_{181}/A\beta_{1–42}$ (p=0.47) and visuospatial functioning. However, analyses may be underpowered as visuospatial scores were only available for a subset of N=29 participants.

Supplementary Table 13. AD-metaROI cortical MRI mediators between CSF markers and ADSP-PHC Composite Memory. Only AD-metaROI measures associated with CSF biomarkers in primary analyses were evaluated; we corrected for multiple comparisons across 13 mediation tests.

CSF	MRI Model	Cortical Mediator	Direct Effect		Mediation Effect		
			β	P	β	P	%
**Aβ** *p*=0.03 *β*=0.23	MT-NODDI	ICVF	0.15	0.17	0.08	0.04	33.6
		ODI	0.23	0.054	0.01	0.74	4.3
	MAP-MRI	QIV	0.14	0.22	0.10	**0.008****	41.7
**$\log_{10}$ pTau** *p*=0.03 *β*=-0.27	FreeSurfer	CTh	-0.10	0.46	-0.17	**<$2x10^{-16}$****	64.1
**pTau/Aβ** *p*=0.0015 *β*=-0.37	MT-NODDI	ICVF	-0.29	**0.012****	-0.08	0.078	21.0
		ISOVF	-0.23	0.04	-0.13	**0.008****	36.9
	MAP-MRI	MSD	-0.28	**0.014****	-0.08	0.046	22.6
		QIV	-0.25	**0.026****	-0.11	0.02	31.0
		RTAP	-0.31	**0.01****	-0.06	0.10	16.6
		RTOP	-0.32	**0.006****	-0.05	0.19	13.2
		RTPP	-0.29	**0.014****	-0.08	0.1	20.8
	DTI	MD	-0.27	**0.018****	-0.10	0.03	26.6
	FreeSurfer	CTh	-0.20	0.12	-0.17	0.02	45.3

**p<FDR Threshold for 13 tests; Direct $p \leq 0.026$; Mediation $p \leq 0.008$

2.12 Spatially distinct patterns of ICVF and ISOVF associations with amyloid

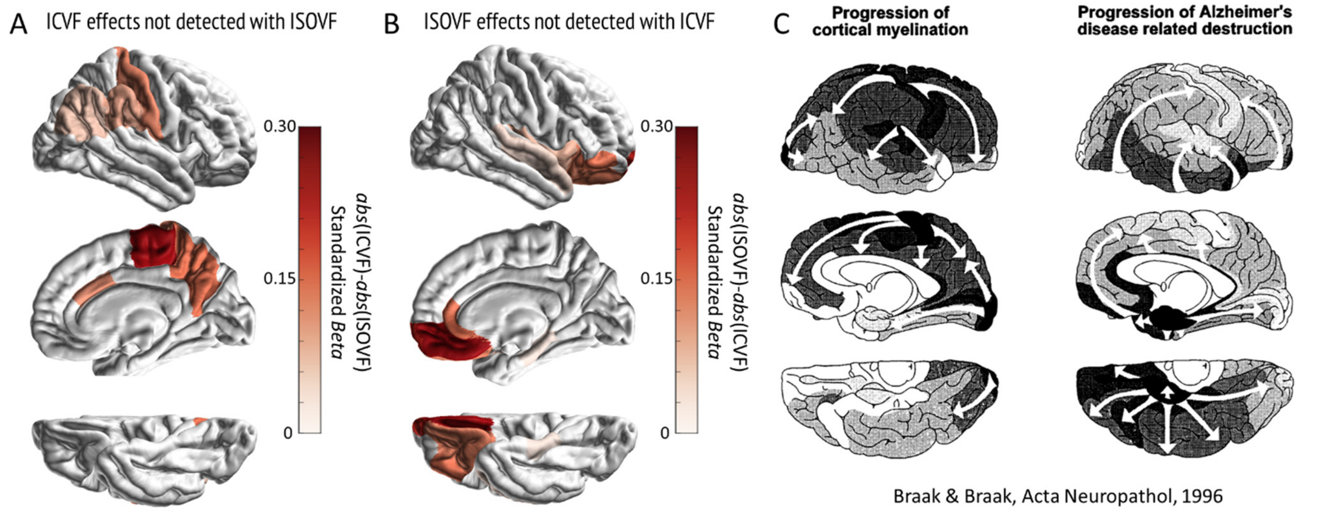

Supplementary Figure 17. In all regions that were significantly associated with amyloid for either ICVF or ISOVF, but not both, we took the difference in magnitude of the effect sizes to capture the diverging patterns of effects. (**A**) In regions where only ICVF associations were significant, the absolute value of the ICVF effect minus the absolute value of the ISOVF effect revealed the largest differences in early/highly myelinated sensory-motor cortical regions like the paracentral cortex. (**B**) In regions where only ISOVF associations were significant, absolute ISOVF minus absolute ICVF effects revealed the largest differences in late/lightly myelinated cortical regions like the medial orbitofrontal cortex. (**C**) These dMRI measures may capture the proposed inverse relationship between the pattern of pathology progression and myelination during development (13, 14). We received permission from Springer Nature Copyright Clearance Center (license number: 5661630852672) to reprint the figure in C from "Development of Alzheimer-related neurofibrillary changes in the neocortex inversely recapitulates cortical myelogenesis," by Braak and Braak, 1996, *Acta Neuropathologica*, 92(2), p. 197–201.

2.13 CSF pTau and delayed working memory

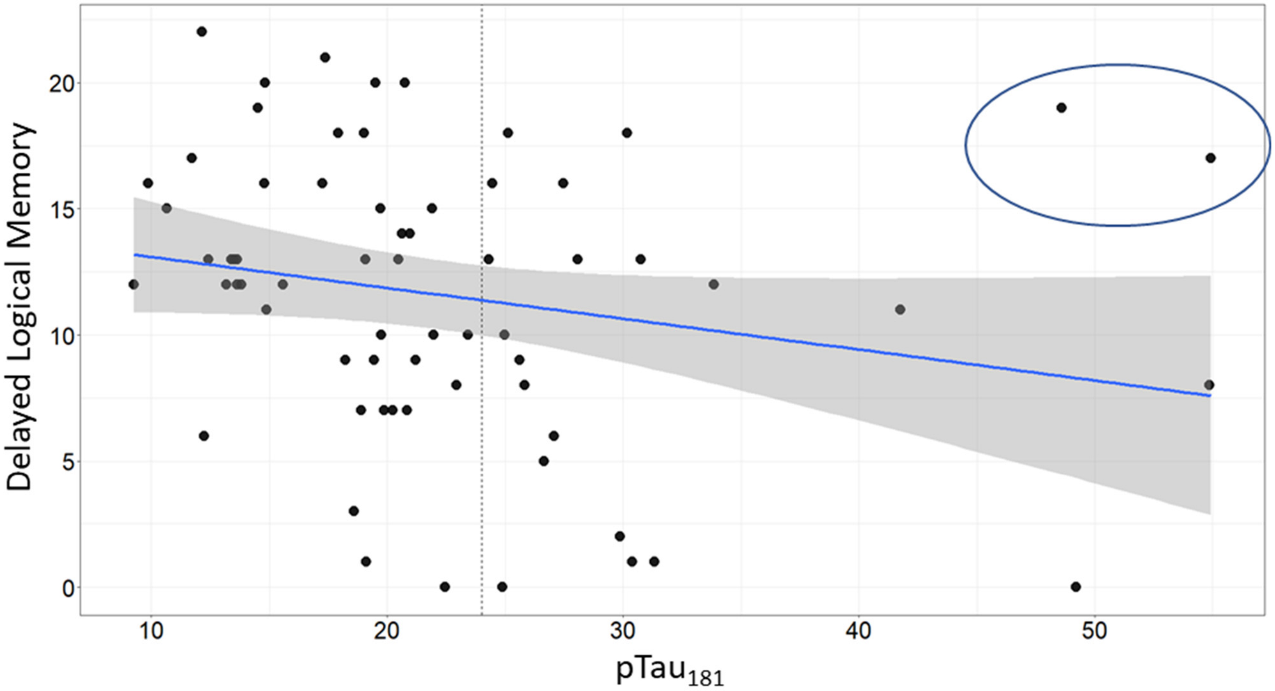

Supplementary Figure 18. The lack of significant associations between CSF pTau and delayed memory may be due in part to the limited number of participants in our study, particularly those with high pTau concentrations. Two CU individuals (one Aβ+ and one Aβ-) with high delayed memory scores and high pTau were sufficient to drive the association (or lack thereof).

3. Supplementary References

1. Ozarslan E, Koay CG, Shepherd TM, Komlosh ME, Irfanoglu MO, Pierpaoli C, et al. Mean apparent propagator (MAP) MRI: a novel diffusion imaging method for mapping tissue microstructure. Neuroimage. 2013;78:16-32.
2. Baxi M, Cetin-Karayumak S, Papadimitriou G, Makris N, van der Kouwe A, Jenkins B, et al. Investigating the contribution of cytoarchitecture to diffusion MRI measures in gray matter using histology. Front Neuroimaging. 2022;1:947526.
3. Wu YC, Field AS, Alexander AL. Computation of diffusion function measures in q-space using magnetic resonance hybrid diffusion imaging. IEEE Trans Med Imaging. 2008;27(6):858-65.
4. Wang P, He J, Ma X, Weng L, Wu Q, Zhao P, et al. Applying MAP-MRI to Identify the WHO Grade and Main Genetic Features of Adult-type Diffuse Gliomas: A Comparison of Three Diffusion-weighted MRI Models. Acad Radiol. 2023;30(7):1238-46.
5. Fick R, Pizzolato M, Wassermann D, Zucchelli M, Menegaz G, Deriche R. A sensitivity analysis of Q-space indices with respect to changes in axonal diameter, dispersion and tissue composition2016.
6. Zucchelli M, Brusini L, Andres Mendez C, Daducci A, Granziera C, Menegaz G. What lies beneath? Diffusion EAP-based study of brain tissue microstructure. Med Image Anal. 2016;32:145-56.
7. Baxi M, Ning L, Cetin-Karayumak S, Kubicki M, Rathi Y. Validation of Diffusion Propagator Imaging measures in White Matter using Histology. In Proc Intl Soc Mag Reson Med. 2020;28.
8. Folstein MF, Folstein SE, McHugh PR. "Mini-mental state". A practical method for grading the cognitive state of patients for the clinician. J Psychiatr Res. 1975;12(3):189-98.
9. Berg L. Clinical Dementia Rating (CDR). Psychopharmacol Bull. 1988;24(4):637-9.
10. Parker TD, Slattery CF, Zhang J, Nicholas JM, Paterson RW, Foulkes AJM, et al. Cortical microstructure in young onset Alzheimer's disease using neurite orientation dispersion and density imaging. Hum Brain Mapp. 2018;39(7):3005-17.
11. Vogt NM, Hunt JF, Adluru N, Dean DC, Johnson SC, Asthana S, et al. Cortical Microstructural Alterations in Mild Cognitive Impairment and Alzheimer's Disease Dementia. Cerebral cortex (New York, NY : 1991). 2020;30(5):2948-60.
12. Sexton CE, Kalu UG, Filippini N, Mackay CE, Ebmeier KP. A meta-analysis of diffusion tensor imaging in mild cognitive impairment and Alzheimer's disease. Neurobiol Aging. 2011;32(12):2322 e5-18.
13. Braak H, Braak E. Development of Alzheimer-related neurofibrillary changes in the neocortex inversely recapitulates cortical myelogenesis. Acta Neuropathol. 1996;92(2):197-201.
14. Bartzokis G, Lu PH, Mintz J. Human brain myelination and amyloid beta deposition in Alzheimer's disease. Alzheimers Dement. 2007;3(2):122-5.
